# Rapid Automated Quantification of Triacylglyceride Crystallinity in Molecular Dynamics Simulations

**DOI:** 10.1021/acs.jcim.2c00972

**Published:** 2022-11-04

**Authors:** Robert J. Cordina, Beccy Smith, Tell Tuttle

**Affiliations:** † Mondele̅z UK R&D Ltd., PO Box 12, Bournville Lane, Birmingham B30 2LU, U.K.; ‡ Department of Pure and Applied Chemistry, University of Strathclyde, 295 Cathedral Street, Glasgow G1 1XL, U.K.

## Abstract

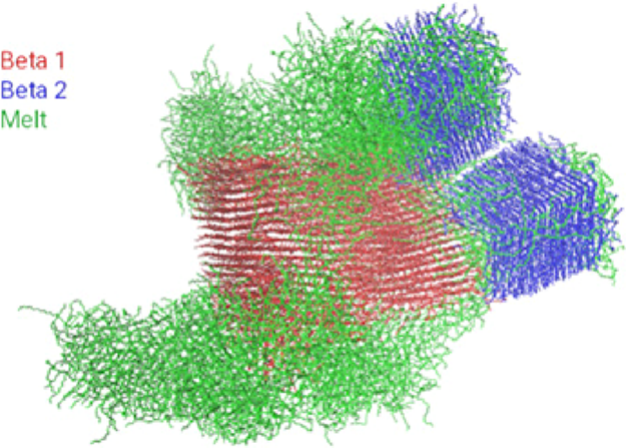

The relative stability of crystalline polymorphs and the transition between crystalline and melt phases are key parameters in determining the physical properties of triacylglycerides used in food. However, while the determination of properties experimentally is well-defined, the ability to predict the onset of melting and discriminate between polymorphs is less well-defined within a molecular dynamics simulation environment. In this work, we present metrics for measuring the crystallinity, including a new metric, the near-neighbor occupancy time, giving a rapid determination of how many, and which, molecules are found in a crystal over a simulation trajectory, and the polymorphic determination of triacylglycerides over a simulation trajectory.

## Introduction

The phase transitions (from melt to crystalline and vice-versa, as well as between different polymorphs) of triacylglycerides (TAGs) are an area of intense research.^1−10^ Knowledge of these physical properties, and how and when they occur, is important, as by knowing this they can then also be controlled. One of the major challenges in studying these transitions computationally is the difficulty with determining and quantifying the boundary between different phases as well as between different polymorphs.

In a 2011 paper by Brasiello *et al.*,^1^ the phase change from liquid to solid is determined by the increase in the density of the system, while the degree of crystallinity is determined by two *ad hoc* parameters, defined by the authors as planarity (a measure of how planar the fatty acid chains are in any given TAG molecule) and conformational parameter (a measure of the chair, tuning-fork or trident shape of the TAG molecules). These two parameters, along with a visual determination of the crystalline molecular configuration, were used to determine the polymorph of the crystallized system (in this case, the α polymorph). Pizzirusso *et al.*(5,6) use the same parameters to determine phase change and molecular conformation, as well as the liquid-crystalline orientation parameter *P*_2_ and the order matrix **R**(11−14) to quantify the order of the crystalline state. *P*_2_ gives a measure of the alignment of a given axis along a molecule with respect to a set direction (the director), where higher *P*_2_ values are indicative of crystalline packing.^6^ None of these studies^1,5,6^ however provide a means of differentiating between polymorphs.

Different polymorphs are a result of a different packing configuration of the same molecules, resulting in different densities and lattice measurements, namely, the unit cell’s dimensions and the α, β, and γ angles. In most cases, however, these measurements are too similar to be of use to distinguish between them effectively.^15^ This is also the case for the β_1_ and β_2_ polymorphs of TAGs.^16,17^ Given this, the actual orientation in space of the molecules has to be determined, such as by X-ray diffraction. The differences in molecular orientation are not generally used as a metric when different polymorphs are simulated using molecular dynamics. Irrespective of whether the simulations are carried out on organic or inorganic compounds, this is generally done on a single polymorph to confirm whether the lattice measurements obtained after equilibration are comparable to those obtained empirically, *i.e.*, as an evaluation of the suitability of the force field being used.^6,18−20^

In this work, multiple approaches to determining and quantifying the solid–liquid transition, as well as the polymorph differentiation of mono-unsaturated TAGs, are presented. This includes the determination of the presence, or otherwise, of different polymorphs in the same simulation box. While the development of these metrics was carried out on a united atom representation of 1-palmitoyl-2-oleoyl-3-stearoyl-*sn*-glycerol (*sn*-POSt), the same principles can be applied to any similar molecules with appropriate modification of the analysis algorithm, namely, the definition of the atoms or atoms to be used for any distance and angle determination from the simulation trajectory.

## Computational Methods

**Figure 1:**
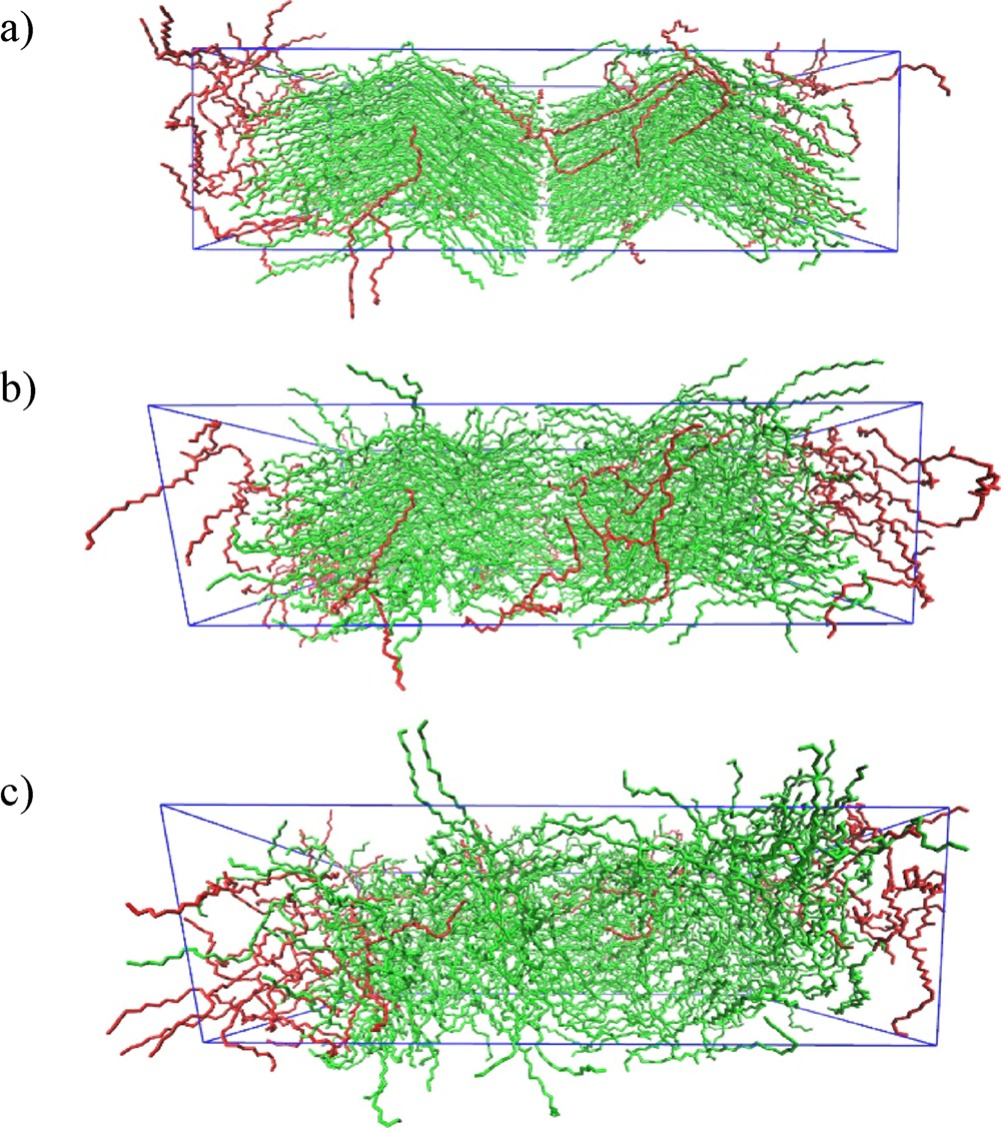
Snapshots of the simulation box. The green and red molecules indicate those molecules that were initially crystalline and positioned randomly, respectively. (a) Minimized molecules (0 ns); (b) crystal melting at 27 ns; (c) fully melted crystal at 50 ns.

The applicability of the developed metrics is illustrated through the equilibration of a box containing only *sn*-POSt molecules. The first simulation box contained 168 molecules (7 × 1 × 6 unit cells, with each unit cell having four molecules) in a single β_2_ polymorph crystal and 32 molecules placed randomly around the crystal, giving, at the starting point, 84% of the molecules which are crystalline and 16% which are not (Figure 1). The system was equilibrated using the modified NERD force field,^21^ with the first 25 ns at 273 K, after which the temperature was raised to 373 K over 10 ps, and held at that temperature up to a total of 50 ns.

The second simulation box contained 1200 molecules, of which 400 (33.3%) were in a single β_1_ crystal (10 × 1 × 10 unit cells) and 200 (16.67%) were in a single β_2_ crystal (10 × 1 × 5 unit cells), while the remaining 600 molecules were placed randomly in the simulation box.

All the simulations were carried out using GROMACS,^22,23^ while the analysis and calculations described below were implemented in Python 3.6. The implementation of calculations on the graphics processing unit (GPU) for the determination of the polymorphs was done *via* the use of the numba module.^24^

All equilibrations were carried out using an isobaric/isothermal (NPT) ensemble, using a v-rescale thermostat and a Berendsen barostat. Constant atmospheric pressure (1.01325 bar) was maintained by using anisotropic pressure coupling, with a compressibility of 1 × 10^–5^ bar^–1^ in the *x*, *y*, and *z* directions. Temperature coupling was set at 1 ps, while pressure coupling was set at 10 ps. A time step of 2 fs was used for all equilibrations. The cut-off scheme was set to Verlet, with the Coulomb and vdW cut-off distances set to 1.1 nm and with the vdW-modifier being set to potential-shift. The coulombtype was set to particle-mesh Ewald, while no epsilon-rf value was specified.

## Results and Discussion

### Determination of Crystallinity

**Figure 2:**
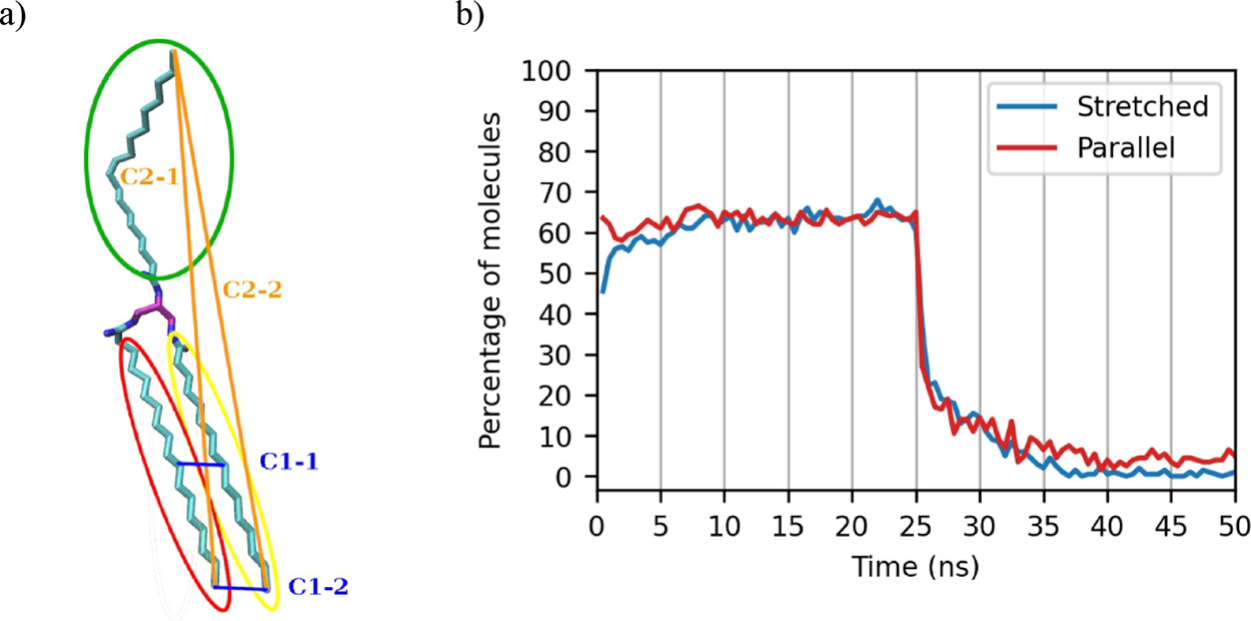
(a) United atom representation of *sn*-POSt (palmitic (at *sn*-1 position) = yellow circle, oleic (*sn*-2) = green circle, and stearic (*sn*-3) = red circle). Blue lines = distances measured for parallel calculation (C1–*x*). Orange lines = distances measured for stretched determination (C2–*x*). (b) Overlaid plots of the percentage of molecules in the simulation box which are fully “stretched” (blue) and have the *sn*-1 and *sn*-3 chains parallel (red) versus time.

The first two metrics used are intramolecular measurements determining the distances between specific atoms. A fully crystalline TAG such as *sn*-POSt has a specific conformation, irrespective of the polymorph in which it is in, where the palmitic and stearic chains at the *sn*-1 and *sn*-3 positions respectively are parallel to each other, while the oleic chain at the *sn*-2 position is positioned away from the other two chains (Figure 2a).^16,17^ The first metric is thus the determination of the distance between two sets of atoms on the palmitic and stearic chains (two on each chain), (C1–*x*). Two distances are required as this is an indication that the two chains are parallel, and not simply randomly close, which could be the case if only one interchain distance had to be used. In this case, the distances used were the last atoms of both chains (in this case, the sixteenth palmitic chain atom and eighteenth stearic chain atom) and two mid-chain atoms (the ninth and eleventh atoms on the palmitic and stearic chains, respectively) (Figure 2a—blue lines). These were chosen due to the very flexible nature of these chains, and hence, satisfying both distance criteria is a clear indication that the chains are close and parallel.

The second intramolecular metric is a measure of the distance between the last oleic chain atom and the last palmitic and stearic oleic chain atoms, *i.e.*, spanning the length of the molecule (Figure 2a—orange lines) (C2–*x*). In this case, the molecule was determined to fit the definition if the oleic-to-stearic (C2–1) and oleic-to-palmitic (C2–2) distances were met. In all cases, the distance criteria were determined to be met if the distances were found to be at the reference value ± two standard deviations. The reference value and standard deviation only need to be determined once. This is done by determining the distances of the relevant atom pairs from all molecules of a perfect crystal of an equilibrated system over a few nanoseconds. The reference value is taken to be the average distance for that particular atom pair.

If a molecule such as *sn*-POSt satisfies both distance criteria, then it is “locked” in a crystal and not in a melted phase where the molecule’s chains are free to rotate. A plot of the percentage of molecules which fit these two criteria is shown in Figure 2b, where the melting of the crystal is very clear by the sharp drop in the molecules which are found to be “parallel” or “stretched”. On melting, the number of molecules which are “stretched” and “parallel” drops from around 65% to nearly 0%. While the initial crystal made up 84% of all the molecules in the simulation box, these two metrics only determined 65% to be “stretched” and “parallel”. On visualizing the trajectory, this discrepancy was determined to be due to the greater degree of freedom of the fatty acid chains of the molecules at the surface of the crystal, even if the molecules as a whole were still part of the crystal.

**Figure 3:**
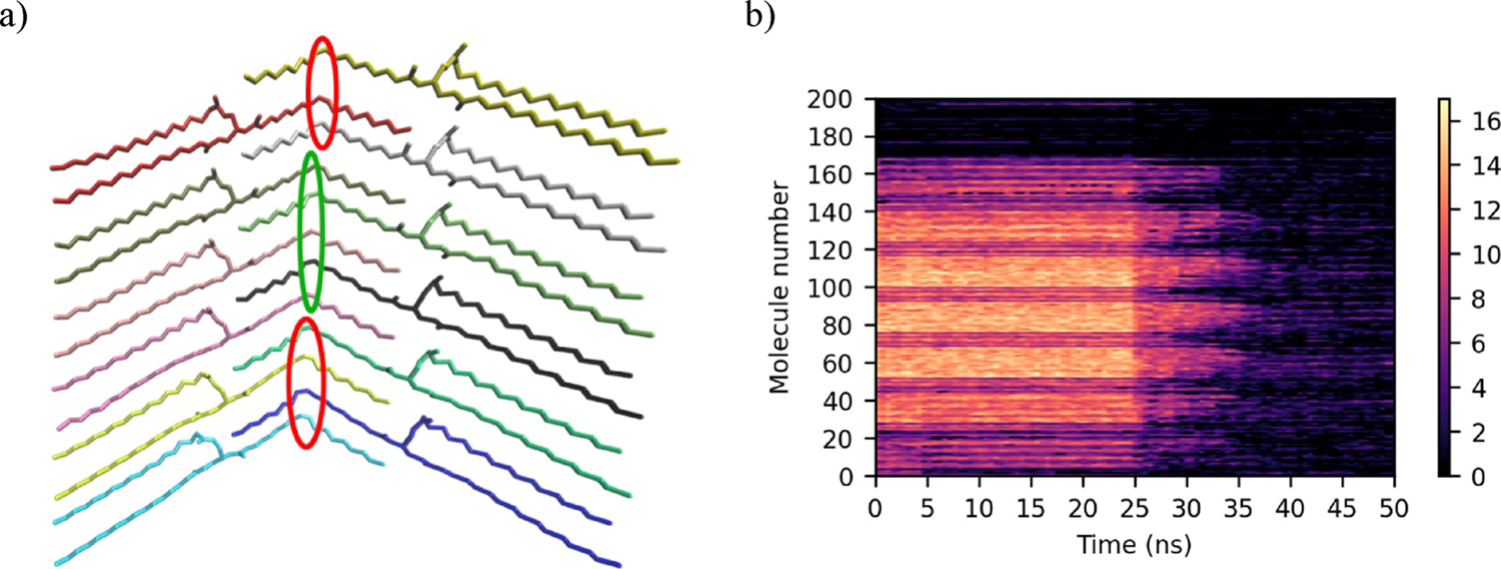
(a) Stacked *sn-*POSt molecules. The central oleic chain atoms circled in green are within the NNO distance of the central pale pink atom. The atoms circled in red are outside the NNO distance with respect to the same atom. (b) NNO plot. A brighter color shows that the same molecules are found within the cut-off distance for any given molecule between subsequent frames.

While the intramolecular metrics defined above highlight where a significant shift in the overall crystal structure occurs, they are also dependent on the conformation of the molecules that constitute the crystal. Therefore, we sought to define a more general metric that could be more widely applied for measuring the degree of crystallinity in a simulation—the near-neighbor occupancy (NNO). We define the NNO as the number of common molecules (*n*) which are within a specified cut-off distance (*x*) of any given molecule (*i*) between subsequent MD trajectory frames. Given that the TAG’s molecular structure is elongated and not a single point, the distance between molecules was defined as the distance between the middle oleic chain atoms, where the cut-off distance (*x*) was set to 1 nm. Hence, if any molecule (or, more specifically, the middle oleic atom of a molecule) was found to be within 1 nm of (the middle oleic atom of) molecule *i* (Figure 3a) in subsequent frames, then this was added to the count for molecule *i*. Plotting the results as a heat map (Figure 3b), where the NNO for each molecule is plotted as a horizontal line with time on the abscissa, provides an excellent visual representation of the crystallinity of the whole system, with the state of each molecule being clearly visible. The “molecule number” on the ordinate of Figure 3b is an index number given to each molecule when the simulation box was generated. In this case, molecules 1–168 are the crystalline molecules, while molecules 169–200 are positioned randomly. With respect to the crystal, molecules 1–28, 45–52, 69–76, 93–100, 117–124, and 141–168 are at the crystal surface, while all other molecules are in the center of the crystal.

When a molecule is in a crystal, the NNO is high, hence shown as brightly colored in the plot, as the molecule and its surrounding molecules are not free to move around. When the crystal melts out, the NNO number drops as the molecules are free to move around the simulation box, and hence, the number of the same molecules being within the cut-off distance of any given molecule between trajectory frames is low. As with the intramolecular measures, this approach clearly indicates that melting of the crystal starts at 25 ns and continues for 10 ns, mirroring the results shown in Figure 2b very closely. In addition to the onset of melting, this metric also provides information on the proportion of molecules which are melted or crystalline and which specific molecules are melted or crystalline. This metric is very sensitive, and it can clearly be observed which molecules are at the crystal faces, with the NNO for those molecules being around 4–6, while those molecules at the center of the crystal have an NNO of 14–16. The molecules with indices 169 to 200 (*i.e.*, the molecules which were added at random positions in the simulation box) show a very low NNO throughout the simulation, indicating their distance from other molecules.

### Determination of Polymorphs

**Figure 4:**
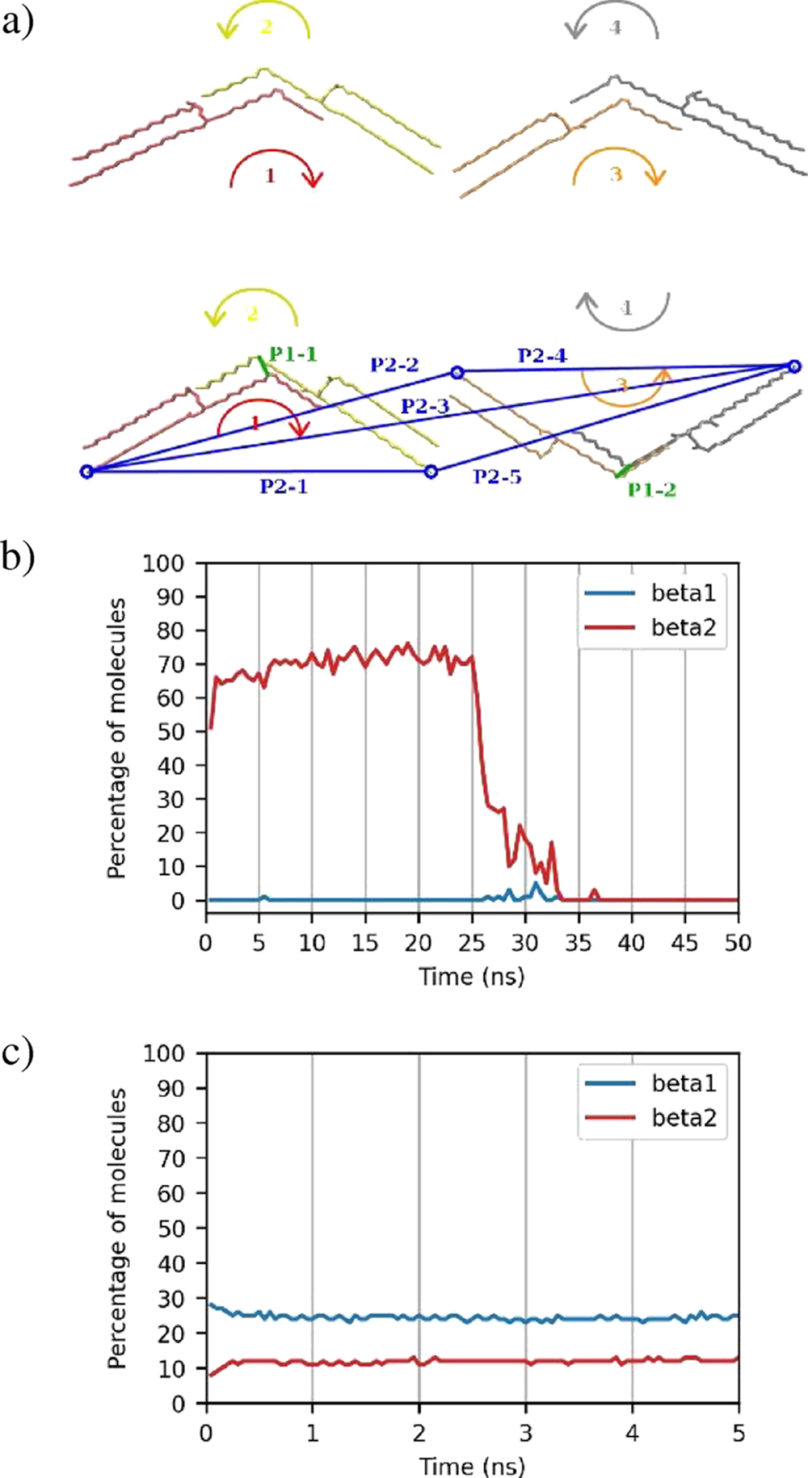
(a) Top: β_2_ polymorph, bottom: β_1_ polymorph of *sn*-POSt. Green (P1–*x*) and blue (P2–*x*) lines = distances measured to determine the proximity of four molecules in a polymorph. Curved arrows = “rotation” of the oleic chain. (b) Plot of the percentage of molecules versus time in the first simulation box containing molecules in a β_2_ crystal (when both polymorphs are shown as 0, this indicates that all the molecules are in the melted phase). (c) Plot of the percentage of molecules versus time in a simulation box containing molecules in both β_2_ and β_1_ polymorphs, with the remainder being melted molecules.

The metrics defined above are a measure of how many molecules are “locked” in an ordered system. They both, however, do not determine if the molecules are in a specific recognized polymorph or, for example, amorphously aggregated. The final developed metric enables the differentiation between polymorphs, exemplified here by differentiating between the β_2_ and β_1_ polymorphs of *sn*-POSt. The approach can however be extended to any other polymorph, or indeed other TAGs, by defining the specific distances for the specific TAGs and their polymorphs. Both the β_2_ and β_1_ polymorphs of *sn*-POSt have unit cells which are defined by four TAG molecules.^16,17^ Their orientation in space is however different (Figure 4a) which allows the independent definition of the two polymorphs differently, based on distances and the orientation of the kinked oleic chain.

Given that it is a four-body problem, multiple distance criteria are required to be met, namely

a) the distance (Figure 4a green lines—P1–*x*) between
  i. molecules 1 (Figure 4a red atoms) and 2 (yellow atoms), (P1–1) and
  ii. molecules 3 (orange atoms) and 4 (gray atoms) (P1–2), *i.e.*, the pairs making up the two “sides” of the unit cell, defined as being the distance between the middle oleic chain atoms

b) the distances between all four molecules, defined as the distance between the last palmitic chain atoms (Figure 4a blue circles and lines—P2–*x*) of molecules
  i. 1 and 2 (P2–1),
  ii. 1 and 3 (P2–2),
  iii. 1 and 4 (P2–3),
  iv. 2 and 4 (P2–4), and
  v. 3 and 4 (P2–5), and

c) P2–1 < P2–2 and P2–4 > P2–5 (this condition obviates the requirement to calculate the distance between molecules 2 and 3).

**Scheme 1:**
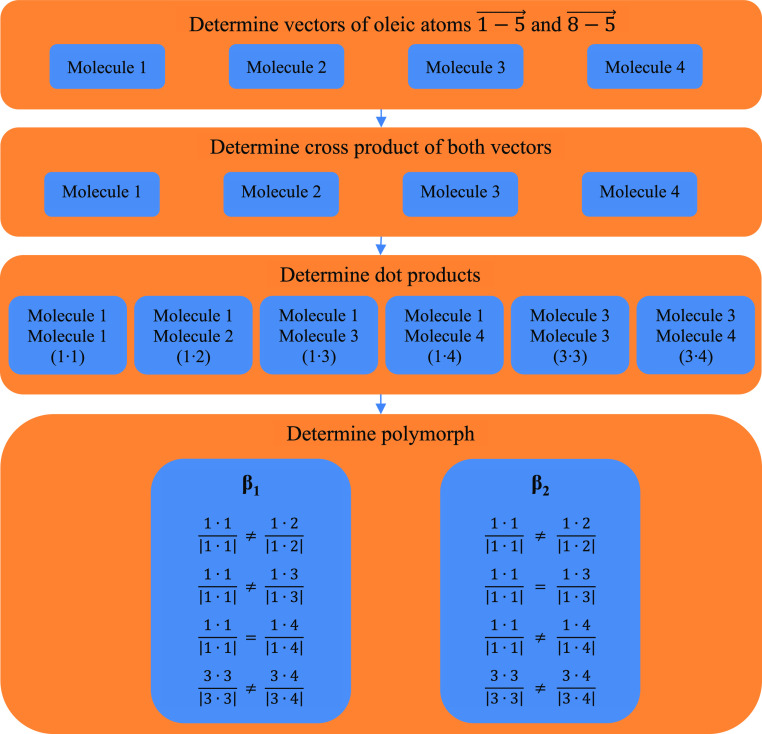
Polymorph Determination Algorithm Based on the Orientation of the Oleic Chain

The distances (P1–*x*, P2–*x*, Figure 4a) do not differ greatly between the two polymorphs, although they are still important to determine to ensure that any four molecules are at the correct distances from each other. The distinguishing element between the two polymorphs is the “direction” of where the oleic kinks are “pointing”. The oleic kinks on both sides of the unit cell of the β_2_ polymorph are pointing in the same direction (Figure 4a top), whereas they point in opposite directions in the β_1_ polymorph (Figure 4a bottom). Differentiation between these polymorphs is carried out as shown in Scheme 1.

The determination of the cross product of the two vectors on any given molecule gives the normal to the plane of the oleic chain (Scheme 1 top two boxes), while the dot product gives the relative rotation of the oleic chain (Scheme 1 third box). To illustrate this, a molecule having an orientation where the first oleic chain atom is to the left, the last oleic chain atom to the right, and the middle oleic chain above might be considered to have a “clockwise” rotation (*e.g.*, Figure 4a top, molecule 1), which gives a positive dot product. Comparing the normal of this molecule with a similarly oriented molecule would give a positive dot product, while comparing the same molecule with an oppositely oriented molecule would give a negative dot product; *i.e.*, the second molecule’s oleic chain rotation is opposite with respect to the first molecule (*e.g.*, Figure 4a top, molecule 2), or, in mathematical terms, the normal to the plane formed by the oleic chain of the second molecule is opposite to that of the first molecule. The last step is to differentiate between the polymorphs. This is done by comparing the various dot products found in the previous step (Scheme 1 bottom box). Given that the different dot products may not have the exact same magnitude, each dot product is divided by its own magnitude to give a “rotation” of +1 or −1, making for easy comparison between different dot products. As can be seen in Scheme 1, the comparison of the various dot products is not the same for the different polymorphs (namely, for 1·1 with 1·3 and 1·1 with 1·4), thus allowing for differentiation between them.

Multibody problems, such as determining whether four molecules are in a unit cell, and if so, in which polymorph, are very computationally expensive, as the number of permutations rises very rapidly with an increasing number of molecules. The number of permutations can be reduced if only four molecules which are near each other are checked at any iteration. Given the elongated dimensions of a unit cell, this is however only applicable if the number of molecules is very high, thus resulting in a large simulation box, as only then will the dimensions of a unit cell be significantly smaller than that of the box. However, even with a relatively small number of molecules, the number of possible permutations is still very large (1000 molecules result in approximately 10^12^ permutations). Due to this, we have implemented the polymorphic determination function to be calculated on a GPU using the numba.cuda module in Python. The numba module allows the function to be compiled before execution, allowing for much faster run times, while the cuda submodule carries out the calculations of the compiled function on the GPU, allowing for a computational wall time decrease of orders of magnitude. When this algorithm was run on the GPU, in this case an Nvidia RTX5000, the computation time was just under 2 min. When the same algorithm was run on a single CPU core, thus computing all possible molecule permutations in sequence, the computation time was around 38 days. This means that GPU implementation is approximately 27,000 times more efficient.

Given the parallelization of GPU calculations and the possibility that any given molecule may be determined to be in a unit cell more than once due to the distance measurement tolerances, as well as allowing for the fact that there may be multiple unit cells which are side by side, it is thus impossible to prevent the possibility that more unit cells are counted than there actually are. To solve for this, a ledger is kept for each molecule to which 1 is added every time that molecule is found to be in one or the other polymorph. At the end, a comparison of the polymorphic counts for each molecule is carried out, and if β_1_ > β_2_ then β_1_ = 1, if β_1_ < β_2_ then β_2_ = 1, if β_1_ = β_2_ = 0 (*i.e.*, a melt) then β_1_ = β_2_ = 0, and in the unlikely event that β_1_ = β_2_, then β_1_ = β_2_ = 0.5. Summing up the number of molecules which have thus been assigned as being in the β_1_ or β_2_ polymorph, one can then easily find the ratio or percentage of molecules which are in either polymorph.

This algorithm was tested on the same simulation trajectory as above, where Figure 4b clearly shows that only the β_2_ polymorph is detected until melting. The second tested system consisted of two crystals in different polymorphs (16.67 and 33.33% of total molecules in the β_2_ and β_1_ polymorphs, respectively), plus a number of randomly positioned molecules (50%). Figure 4c shows the determination of the ratio of the different polymorphs in the simulation box over time and in the correct ratios. The simulation box was equilibrated for only 5 ns as this system was only used to highlight the ability to differentiate between the two polymorphs.

## Conclusions

In conclusion, we have demonstrated multiple approaches to determine crystallinity. The intramolecular measurements to determine the parallel configuration of the *sn*-1 and *sn*-3 chains and the stretched configuration of the *sn*-1 and *sn*-2 and *sn*-2 and *sn*-3 chains are computationally cheap; however, they must be interpreted together while also not giving any information as to whether a molecule is in a unit cell or in a specific polymorph. The NNO metric is also computationally cheap and thus gives a very rapid determination of how many, and which, molecules are in a crystal. It is not, however, able to characterize the polymorph in which the molecules are in. The polymorph determination algorithm is a powerful and flexible method, which is, however, computationally expensive due to the large number of possible four-body permutations, and which thus requires GPU implementation. While all of these metrics have been applied to *sn*-POSt, these can be easily applied to similar TAGs by simply changing the reference distances.

## Supporting Information Available

Full Python analysis script (PDF)

## Notes

All data underpinning this publication are openly available from the University of Strathclyde KnowledgeBase at https://doi.org/10.15129/f711eca0-b564-43ec-8c6e-7178ba78f4eb.
