# Supplementary material for: Rapid Automated Quantification of Triacylglyceride Crystallinity in Molecular Dynamics Simulations
Source: J Chem Inf Model. 2022 Nov 4;62(22):5601–6. doi: 10.1021/acs.jcim.2c00972 (PMC9709910; doi:10.1021/acs.jcim.2c00972)
Supplement: Supplementary file 1 — ci2c00972_si_001.pdf [file ci2c00972_si_001.pdf]

Supporting Information for: Rapid Automated Quantification of Triacylglyceride Crystallinity in Molecular Dynamics Simulations

Robert J. Cordina[1,2], Beccy Smith[1], Tell Tuttle[2,]*

[1] Mondelēz UK R&D Ltd., PO Box 12, Bournville Lane, Birmingham B30 2LU, UK.

[2] Department of Pure and Applied Chemistry, University of Strathclyde, 295 Cathedral Street, Glasgow G1 1XL, UK.

* tell.tuttle@strath.ac.uk

Python analysis script

```
#!/usr/bin/env python3
# -*- coding: utf-8 -*-

import time
import re
import math
import numpy as np

import matplotlib.pyplot as plt
from mpl_toolkits.axes_grid1 import make_axes_locatable

from numba import jit as njit
from numba import prange
from numba import cuda

from mdtraj import load_xtc as xtc



def tag_phases_specifics(TAG, phase):

    """
    trajectory has been converted using gmx trjconv (using -ur compact -pbc mol) to
    avoid molecules with atoms on different sides of the simulation box due to periodicity
    """
    min_file = 'GROMACS_minimised_box.gro'
    traj_file = 'GROMACS_trajectory_file.xtc'
    log_file = 'GROMACS_trajectory_log.log'


    """
    Specifying atom index numbers in each molecule,
    subtracting 1 from all atom numbers to compensate for python 0-indexing.
    These need to be changed for each molecule.
    xy = integer
    x.abcd = float in nm
    """
    if TAG == 'XXX':
        # number of atoms in each molecule
        atom_count = xy
        # middle and end atom indices in sn-1 chain
        sn1_mid = xy-1
        sn1_end = xy-1
        # first, middle, and end atom indices in sn-2 chain
        sn2_fir = xy-1
        sn2_mid = xy-1
        sn2_end = xy-1
        # middle and end atom indices in sn-3 chain
        sn3_mid = xy-1
        sn3_end = xy-1

        # sn-1 to sn-3 end atom reference distance and 2 * standard deviation value - used for "parallel" molecule chain determination
        sn1end_sn3end_dist_avg = x.abcd
        sn1end_sn3end_2stdev = x.abcd

        # sn-1 to sn-3 mid atom reference distance and 2 * standard deviation value - used for "parallel" molecule chain determination
        sn1mid_sn3mid_dist_avg = x.abcd
        sn1mid_sn3mid_2stdev = x.abcd

        # sn-2 to sn-1 end atom reference distance and 2 * standard deviation value - used for "stretched" molecule chain determination
        sn2end_sn1end_dist_avg = x.abcd
        sn2end_sn1end_2stdev = x.abcd

        # sn-2 to sn-3 end atom reference distance and 2 * standard deviation value - used for "stretched" molecule chain determination
        sn2end_sn3end_dist_avg = x.abcd
        sn2end_sn3end_2stdev = x.abcd

        # sn-1 chain end atom reference distances for molecules 1-2, 1-3, 1-4, 3-4 and 2-4 and 2 * standard deviation - used for polymorphic determination
        one_sn1_end_to_two_sn1_end_avg = x.abcd
        one_sn1_end_to_three_sn1_end_avg = x.abcd
        one_sn1_end_to_four_sn1_end_avg = x.abcd
        three_sn1_end_to_four_sn1_end_avg = x.abcd
        two_sn1_end_to_four_sn1_end_avg = x.abcd
```

```
    one_sn1_end_to_two_sn1_end_2stdev = x.abcd
    one_sn1_end_to_three_sn1_end_2stdev = x.abcd
    one_sn1_end_to_four_sn1_end_2stdev = x.abcd
    three_sn1_end_to_four_sn1_end_2stdev = x.abcd
    two_sn1_end_to_four_sn1_end_2stdev = x.abcd


  return min_file, traj_file, log_file, atom_count, sn1_mid, sn1_end, sn2_fir, sn2_mid, sn2_end, sn3_mid, sn3_end, sn1end_sn3end_dist_avg, sn1end_sn3end_2stdev, sn1mid_sn3mid_dist_avg, sn1mid_sn3mid_2stdev, sn2end_sn1end_dist_avg, sn2end_sn1end_2stdev, sn2end_sn3end_dist_avg, sn2end_sn3end_2stdev, one_sn1_end_to_two_sn1_end_avg, one_sn1_end_to_three_sn1_end_avg, one_sn1_end_to_four_sn1_end_avg, three_sn1_end_to_four_sn1_end_avg, two_sn1_end_to_four_sn1_end_avg, one_sn1_end_to_two_sn1_end_2stdev, one_sn1_end_to_three_sn1_end_2stdev, one_sn1_end_to_four_sn1_end_2stdev, three_sn1_end_to_four_sn1_end_2stdev, two_sn1_end_to_four_sn1_end_2stdev


@njit(nopython=True, parallel=True)
def stretch_and_parallel(stretch_percent,
                         parallel_percent,
                         sn2_end_to_sn1_end_dist,
                         sn2_end_to_sn3_end_dist,
                         sn1_end_to_sn3_end_dist,
                         sn1_mid_to_sn3_mid_dist,
                         sn2_end_to_sn1_end_count,
                         sn2_end_to_sn3_end_count,
                         coordinates,
                         sn1_mid, sn1_end,
                         sn2_fir, sn2_mid, sn2_end,
                         sn3_mid, sn3_end,
                         sn1end_sn3end_dist_avg, sn1end_sn3end_2stdev,
                         sn1mid_sn3mid_dist_avg, sn1mid_sn3mid_2stdev,
                         sn2end_sn1end_dist_avg, sn2end_sn1end_2stdev,
                         sn2end_sn3end_dist_avg, sn2end_sn3end_2stdev,
                         atom_count):
  """
  Function first calculating the four different intramolecular distances required to determine if a
  molecule has the chains at the sn-1 and sn-3 positions parallel (sn1_end-sn3_end and sn1_mid-sn3_mid), and if the
  chains at sn-2+sn-1 (sn2_end-sn1_end) and sn-2+sn-1 (sn2_end-sn3_end) are fully stretched, and then determining
  whether the molecules are indeed stretched and have parallel chains.

  This is calculated for each molecule in each trajectory frame.

  The njit decorator with nopython=True allows for the function to be compiled and thus execute faster.
  The argument parallel=True instructs for the calulations to be carried out in parallel over all the CPU
  cores available. This must be coded in conjunction with prange in the for loops.

  """

  for frame in prange(num_frames):
    for num in prange(mol_count):

      sn2_end_to_sn1_end_dist[frame,num] = ((coordinates[frame,sn2_end+num*atom_count,0] - coordinates[frame,sn1_end+num*atom_count,0])**2 +
                  (coordinates[frame,sn2_end+num*atom_count,1] - coordinates[frame,sn1_end+num*atom_count,1])**2 +
                  (coordinates[frame,sn2_end+num*atom_count,2] - coordinates[frame,sn1_end+num*atom_count,2])**2) ** 0.5

      sn2_end_to_sn3_end_dist[frame,num] = ((coordinates[frame,sn2_end+num*atom_count,0] - coordinates[frame,sn3_end+num*atom_count,0])**2 +
                  (coordinates[frame,sn2_end+num*atom_count,1] - coordinates[frame,sn3_end+num*atom_count,1])**2 +
                  (coordinates[frame,sn2_end+num*atom_count,2] - coordinates[frame,sn3_end+num*atom_count,2])**2) ** 0.5

      sn1_end_to_sn3_end_dist[frame,num] = ((coordinates[frame,sn1_end+num*atom_count,0] - coordinates[frame,sn3_end+num*atom_count,0])**2 +
                  (coordinates[frame,sn1_end+num*atom_count,1] - coordinates[frame,sn3_end+num*atom_count,1])**2 +
                  (coordinates[frame,sn1_end+num*atom_count,2] - coordinates[frame,sn3_end+num*atom_count,2])**2) ** 0.5

      sn1_mid_to_sn3_mid_dist[frame,num] = ((coordinates[frame,sn1_mid+num*atom_count,0] - coordinates[frame,sn3_mid+num*atom_count,0])**2 +
                  (coordinates[frame,sn1_mid+num*atom_count,1] - coordinates[frame,sn3_mid+num*atom_count,1])**2 +
                  (coordinates[frame,sn1_mid+num*atom_count,2] - coordinates[frame,sn3_mid+num*atom_count,2])**2) ** 0.5

  # calculating min and max tolernace values
  sn2end_sn1end_min = sn2end_sn1end_dist_avg - sn2end_sn1end_2stdev
  sn2end_sn1end_max = sn2end_sn1end_dist_avg + sn2end_sn1end_2stdev
  sn2end_sn3mid_min = sn2end_sn3end_dist_avg - sn2end_sn3end_2stdev
  sn2end_sn3mid_max = sn2end_sn3end_dist_avg + sn2end_sn3end_2stdev

  for frame in range(num_frames):
    stretch_count = 0
    for num in range(mol_count):
```

```
            # calculating the number of instances the extreme atoms are at the specified distance +/- tolerance
            if ((sn2end_sn1end_min <= sn2_end_to_sn1_end_dist[frame,num] <= sn2end_sn1end_max) and
                (sn2end_sn3mid_min <= sn2_end_to_sn3_end_dist[frame,num] <= sn2end_sn3mid_max) ):
                stretch_count += 1

        stretch_percent[frame] = stretch_count/mol_count*100

    # calculating min and max tolernace values
    sn1end_sn3end_min = sn1end_sn3end_dist_avg - sn1end_sn3end_2stdev
    sn1end_sn3end_max = sn1end_sn3end_dist_avg + sn1end_sn3end_2stdev
    sn1mid_sn3mid_min = sn1mid_sn3mid_dist_avg - sn1mid_sn3mid_2stdev
    sn1mid_sn3mid_max = sn1mid_sn3mid_dist_avg + sn1mid_sn3mid_2stdev

    for frame in range(num_frames):
        parallel_count = 0
        for num in range(mol_count):
            # calculating the number of instances the extreme atoms are at the specified distance +/- tolerance
            if ((sn1end_sn3end_min <= sn1_end_to_sn3_end_dist[frame,num] <= sn1end_sn3end_max)  and
                (sn1mid_sn3mid_min <= sn1_mid_to_sn3_mid_dist[frame,num] <= sn1mid_sn3mid_max)) :
                parallel_count += 1

        parallel_percent[frame] = parallel_count/mol_count*100


@njit(nopython=True, parallel=True)
def cutoff_indices_1nm(cutoff_indices,coordinates,sn2_mid,atom_count):
    """
    Function determining which molecules (molecule indices) are within 1 nm away from the reference molecule
    for each molecule in each frame.

    The if statement in the inner for loop avoids the calculation of the distance between the same molecule
    by using the ref != num conditional.

    """

    for frame in prange(num_frames):
        for ref in prange(mol_count):

            # -1 is used in order to start at index 0 when the first molecule within the cut-off distance is found
            element_count = -1
            for num in prange(mol_count):
                if (ref != num and
                     ( ( (coordinates[frame,sn2_mid+ref*atom_count,0] - coordinates[frame,sn2_mid+num*atom_count,0])**2 +
                        (coordinates[frame,sn2_mid+ref*atom_count,1] - coordinates[frame,sn2_mid+num*atom_count,1])**2 +
                        (coordinates[frame,sn2_mid+ref*atom_count,2] - coordinates[frame,sn2_mid+num*atom_count,2])**2) ** 0.5 <= 1) ):

                    # used for indexing
                    element_count += 1
                    # saving the index of the molecule found within the cut-off distance of the reference molecule
                    cutoff_indices[frame, ref, element_count] = num

@njit(nopython=True, parallel=True)
def same_indices(same_indices_count,cutoff_indices):
    """
    Function determining whether a molecule found to be within the cut-off distance of the reference molecule
    is also found within the cut-off distance in the subseqent frame.

    """

    for frame in prange(num_frames):
        for num in prange(mol_count):
            # setting the counter to 0 for each molecule in each frame
            same_indices = 0
            # used to avoid indexing error
            if frame != num_frames-1:
                # element-wise comparison between the indices of the molecules within the cut-off distance for one molecule between successive frames
                # counter is increased by 1 if a molecule satifies the criteria
                for element in cutoff_indices[frame,num,:]:
                    for comparison in cutoff_indices[frame+1,num,:]:
                        if element == comparison and element >= 0 and comparison >= 0:
                            same_indices += 1
                        if same_indices == 0:
                            same_indices_count[frame,num] = 0
                        else:
                            same_indices_count[frame,num] = same_indices

    # setting the values of the last frame equal to that those of the penultimate frame
    same_indices_count[-1,:] = same_indices_count[-2,:]

@cuda.jit
```

```
def unit_cell_determination(frame,
            coordinates,
            flag,
            sn1_end,
            sn2_fir,
            sn2_mid,
            sn2_end,
            atom_count,
            one_to_two_min,
            one_to_two_max,
            one_to_three_min,
            one_to_three_max,
            one_to_four_min,
            one_to_four_max,
            three_to_four_min,
            three_to_four_max,
            two_to_four_min,
            two_to_four_max):
   """
   Function to determine whether four molecules are found in the beta-1 or beta-2 polymorph of sn-POSt.
   The function is executed for only one frame at a time to allow for better memory management.

   The cuda.jit decorator compiles the function and executes it on the GPU.

   """

   # 3D thread positioning on the GPU
   # these are also used to determine the molecule index numbers for three of the four molecule to
   # be used in the polymorph determination algorithm
   x_idx = cuda.blockIdx.x * cuda.blockDim.x + cuda.threadIdx.x
   y_idx = cuda.blockIdx.y * cuda.blockDim.y + cuda.threadIdx.y
   z_idx = cuda.blockIdx.z * cuda.blockDim.z + cuda.threadIdx.z

   # checking array boundaries - ensuring that the thread positioning (and hence the molecule index) is always
   # smaller than the total number of molecules, thus preventing an indexing error
   if (x_idx and y_idx and z_idx) < mol_count:
      # checking that the three molecules indexed by the thread positioning are all different
      if x_idx != (y_idx or z_idx) and y_idx != z_idx:
         # iterating over the other molecules - this acts as the fourth molecule in the polymorph determining algorithm
         for num in range(mol_count):
            # checking that the fourth molecule is not identical to the other three molecules
            if num != (x_idx or y_idx or z_idx):
               mol1, mol2, mol3, mol4 = x_idx, y_idx, z_idx, num


               # general distances check - applicable to both polymorphs
               # calculate subsequent checks only if distances conditionals are met

               if (
                # checking that molecules 1 and 2, and 3 and 4 are close to eachh other
                ( ( (     (coordinates[frame,sn2_mid+mol1*atom_count,0] - coordinates[frame,sn2_mid+mol2*atom_count,0]) ** 2 +
                          (coordinates[frame,sn2_mid+mol1*atom_count,1] - coordinates[frame,sn2_mid+mol2*atom_count,1]) ** 2 +
                          (coordinates[frame,sn2_mid+mol1*atom_count,2] - coordinates[frame,sn2_mid+mol2*atom_count,2]) ** 2 ) ** 0.5 ) < 1  )
                and
                ( ( (     (coordinates[frame,sn2_mid+mol3*atom_count,0] - coordinates[frame,sn2_mid+mol4*atom_count,0]) ** 2 +
                          (coordinates[frame,sn2_mid+mol3*atom_count,1] - coordinates[frame,sn2_mid+mol4*atom_count,1]) ** 2 +
                          (coordinates[frame,sn2_mid+mol3*atom_count,2] - coordinates[frame,sn2_mid+mol4*atom_count,2]) ** 2 ) ** 0.5 ) < 1  )
                and
                # checking distacnes for molecules 1-4, 1-3, 1-2, 2-4 and 3-4
                (  one_to_four_min <
                 ( ( (coordinates[frame,sn1_end+mol1*atom_count,0] - coordinates[frame,sn1_end+mol4*atom_count,0]) ** 2 +
                    (coordinates[frame,sn1_end+mol1*atom_count,1] - coordinates[frame,sn1_end+mol4*atom_count,1]) ** 2 +
                    (coordinates[frame,sn1_end+mol1*atom_count,2] - coordinates[frame,sn1_end+mol4*atom_count,2]) ** 2 ) ** 0.5 ) < one_to_four_max)
                and
                (   one_to_three_min <
                 ( ( (coordinates[frame,sn1_end+mol1*atom_count,0] - coordinates[frame,sn1_end+mol3*atom_count,0]) ** 2 +
                   (coordinates[frame,sn1_end+mol1*atom_count,1] - coordinates[frame,sn1_end+mol3*atom_count,1]) ** 2 +
                   (coordinates[frame,sn1_end+mol1*atom_count,2] - coordinates[frame,sn1_end+mol3*atom_count,2]) ** 2 ) ** 0.5 ) < one_to_three_max
)
                and
                (   one_to_two_min <
                 ( ( (coordinates[frame,sn1_end+mol1*atom_count,0] - coordinates[frame,sn1_end+mol2*atom_count,0]) ** 2 +
                   (coordinates[frame,sn1_end+mol1*atom_count,1] - coordinates[frame,sn1_end+mol2*atom_count,1]) ** 2 +
                   (coordinates[frame,sn1_end+mol1*atom_count,2] - coordinates[frame,sn1_end+mol2*atom_count,2]) ** 2 ) ** 0.5 ) < one_to_two_max  )
                and
                (   two_to_four_min <
                 ( ( (coordinates[frame,sn1_end+mol2*atom_count,0] - coordinates[frame,sn1_end+mol4*atom_count,0]) ** 2 +
                   (coordinates[frame,sn1_end+mol2*atom_count,1] - coordinates[frame,sn1_end+mol4*atom_count,1]) ** 2 +
                   (coordinates[frame,sn1_end+mol2*atom_count,2] - coordinates[frame,sn1_end+mol4*atom_count,2]) ** 2 ) ** 0.5 ) < two_to_four_max  )
                and
```

```
                (   three_to_four_min <
                 ( ( (coordinates[frame,sn1_end+mol3*atom_count,0] - coordinates[frame,sn1_end+mol4*atom_count,0]) ** 2 +
                   (coordinates[frame,sn1_end+mol3*atom_count,1] - coordinates[frame,sn1_end+mol4*atom_count,1]) ** 2 +
                   (coordinates[frame,sn1_end+mol3*atom_count,2] - coordinates[frame,sn1_end+mol4*atom_count,2]) ** 2 ) ** 0.5 ) < three_to_four_max
)
                and
                # checking that molecules 1-2 distance < molecuels 1-3 distance
                (
                   ( (     (coordinates[frame,sn1_end+mol1*atom_count,0] - coordinates[frame,sn1_end+mol2*atom_count,0]) ** 2 +
                          (coordinates[frame,sn1_end+mol1*atom_count,1] - coordinates[frame,sn1_end+mol2*atom_count,1]) ** 2 +
                          (coordinates[frame,sn1_end+mol1*atom_count,2] - coordinates[frame,sn1_end+mol2*atom_count,2]) ** 2 ) ** 0.5 )
                   <
                   ( (     (coordinates[frame,sn1_end+mol1*atom_count,0] - coordinates[frame,sn1_end+mol3*atom_count,0]) ** 2 +
                          (coordinates[frame,sn1_end+mol1*atom_count,1] - coordinates[frame,sn1_end+mol3*atom_count,1]) ** 2 +
                          (coordinates[frame,sn1_end+mol1*atom_count,2] - coordinates[frame,sn1_end+mol3*atom_count,2]) ** 2 ) ** 0.5 ) )
                and
                # checking that molecules 4-2 distance > molecuels 4-3 distance

                (
                   ( (     (coordinates[frame,sn1_end+mol4*atom_count,0] - coordinates[frame,sn1_end+mol2*atom_count,0]) ** 2 +
                          (coordinates[frame,sn1_end+mol4*atom_count,1] - coordinates[frame,sn1_end+mol2*atom_count,1]) ** 2 +
                          (coordinates[frame,sn1_end+mol4*atom_count,2] - coordinates[frame,sn1_end+mol2*atom_count,2]) ** 2 ) ** 0.5 )
                   >
                   ( (     (coordinates[frame,sn1_end+mol4*atom_count,0] - coordinates[frame,sn1_end+mol3*atom_count,0]) ** 2 +
                          (coordinates[frame,sn1_end+mol4*atom_count,1] - coordinates[frame,sn1_end+mol3*atom_count,1]) ** 2 +
                          (coordinates[frame,sn1_end+mol4*atom_count,2] - coordinates[frame,sn1_end+mol3*atom_count,2]) ** 2 ) ** 0.5 ) ) ):


                 # calculate sn2_fir-sn2_mid and sn2_end-sn2_mid vectors for all four molecules
                 # calculated individually due to numba.cuda limitations
                 mol1_85_0 = coordinates[frame,sn2_end+mol1*atom_count,0] - coordinates[frame,sn2_mid+mol1*atom_count,0]
                 mol1_15_0 = coordinates[frame,sn2_fir+mol1*atom_count,0] - coordinates[frame,sn2_mid+mol1*atom_count,0]
                 mol1_85_1 = coordinates[frame,sn2_end+mol1*atom_count,1] - coordinates[frame,sn2_mid+mol1*atom_count,1]
                 mol1_15_1 = coordinates[frame,sn2_fir+mol1*atom_count,1] - coordinates[frame,sn2_mid+mol1*atom_count,1]
                 mol1_85_2 = coordinates[frame,sn2_end+mol1*atom_count,2] - coordinates[frame,sn2_mid+mol1*atom_count,2]
                 mol1_15_2 = coordinates[frame,sn2_fir+mol1*atom_count,2] - coordinates[frame,sn2_mid+mol1*atom_count,2]

                 mol2_85_0 = coordinates[frame,sn2_end+mol2*atom_count,0] - coordinates[frame,sn2_mid+mol2*atom_count,0]
                 mol2_15_0 = coordinates[frame,sn2_fir+mol2*atom_count,0] - coordinates[frame,sn2_mid+mol2*atom_count,0]
                 mol2_85_1 = coordinates[frame,sn2_end+mol2*atom_count,1] - coordinates[frame,sn2_mid+mol2*atom_count,1]
                 mol2_15_1 = coordinates[frame,sn2_fir+mol2*atom_count,1] - coordinates[frame,sn2_mid+mol2*atom_count,1]
                 mol2_85_2 = coordinates[frame,sn2_end+mol2*atom_count,2] - coordinates[frame,sn2_mid+mol2*atom_count,2]
                 mol2_15_2 = coordinates[frame,sn2_fir+mol2*atom_count,2] - coordinates[frame,sn2_mid+mol2*atom_count,2]

                 mol3_85_0 = coordinates[frame,sn2_end+mol3*atom_count,0] - coordinates[frame,sn2_mid+mol3*atom_count,0]
                 mol3_15_0 = coordinates[frame,sn2_fir+mol3*atom_count,0] - coordinates[frame,sn2_mid+mol3*atom_count,0]
                 mol3_85_1 = coordinates[frame,sn2_end+mol3*atom_count,1] - coordinates[frame,sn2_mid+mol3*atom_count,1]
                 mol3_15_1 = coordinates[frame,sn2_fir+mol3*atom_count,1] - coordinates[frame,sn2_mid+mol3*atom_count,1]
                 mol3_85_2 = coordinates[frame,sn2_end+mol3*atom_count,2] - coordinates[frame,sn2_mid+mol3*atom_count,2]
                 mol3_15_2 = coordinates[frame,sn2_fir+mol3*atom_count,2] - coordinates[frame,sn2_mid+mol3*atom_count,2]

                 mol4_85_0 = coordinates[frame,sn2_end+mol4*atom_count,0] - coordinates[frame,sn2_mid+mol4*atom_count,0]
                 mol4_15_0 = coordinates[frame,sn2_fir+mol4*atom_count,0] - coordinates[frame,sn2_mid+mol4*atom_count,0]
                 mol4_85_1 = coordinates[frame,sn2_end+mol4*atom_count,1] - coordinates[frame,sn2_mid+mol4*atom_count,1]
                 mol4_15_1 = coordinates[frame,sn2_fir+mol4*atom_count,1] - coordinates[frame,sn2_mid+mol4*atom_count,1]
                 mol4_85_2 = coordinates[frame,sn2_end+mol4*atom_count,2] - coordinates[frame,sn2_mid+mol4*atom_count,2]
                 mol4_15_2 = coordinates[frame,sn2_fir+mol4*atom_count,2] - coordinates[frame,sn2_mid+mol4*atom_count,2]

                 # calculate cross products - calculated step-wise due to numba.cuda limitations
                 cross_mol1_0 = mol1_85_1*mol1_15_2 - mol1_85_2*mol1_15_1
                 cross_mol2_0 = mol2_85_1*mol2_15_2 - mol2_85_2*mol2_15_1
                 cross_mol3_0 = mol3_85_1*mol3_15_2 - mol3_85_2*mol3_15_1
                 cross_mol4_0 = mol4_85_1*mol4_15_2 - mol4_85_2*mol4_15_1

                 cross_mol1_1 = mol1_85_2*mol1_15_0 - mol1_85_0*mol1_15_2
                 cross_mol2_1 = mol2_85_2*mol2_15_0 - mol2_85_0*mol2_15_2
                 cross_mol3_1 = mol3_85_2*mol3_15_0 - mol3_85_0*mol3_15_2
                 cross_mol4_1 = mol4_85_2*mol4_15_0 - mol4_85_0*mol4_15_2

                 cross_mol1_2 = mol1_85_0*mol1_15_1 - mol1_85_1*mol1_15_0
                 cross_mol2_2 = mol2_85_0*mol2_15_1 - mol2_85_1*mol2_15_0
                 cross_mol3_2 = mol3_85_0*mol3_15_1 - mol3_85_1*mol3_15_0
                 cross_mol4_2 = mol4_85_0*mol4_15_1 - mol4_85_1*mol4_15_0

                 # calculate dot products - calculated step-wise due to numba.cuda limitations
                 dot1_1 = cross_mol1_0*cross_mol1_0 + cross_mol1_1*cross_mol1_1 + cross_mol1_2*cross_mol1_2
                 dot1_2 = cross_mol1_0*cross_mol2_0 + cross_mol1_1*cross_mol2_1 + cross_mol1_2*cross_mol2_2

                 dot1_3 = cross_mol1_0*cross_mol3_0 + cross_mol1_1*cross_mol3_1 + cross_mol1_2*cross_mol3_2
                 dot1_4 = cross_mol1_0*cross_mol4_0 + cross_mol1_1*cross_mol4_1 + cross_mol1_2*cross_mol4_2
```

```
                        dot3_3 = cross_mol3_0*cross_mol3_0 + cross_mol3_1*cross_mol3_1 + cross_mol3_2*cross_mol3_2
                        dot3_4 = cross_mol3_0*cross_mol4_0 + cross_mol3_1*cross_mol4_1 + cross_mol3_2*cross_mol4_2


                        # beta-1 check
                        if (    (dot1_1/abs(dot1_1) != dot1_2/abs(dot1_2))
                            and (dot1_1/abs(dot1_1) != dot1_3/abs(dot1_3))
                            and (dot1_1/abs(dot1_1) == dot1_4/abs(dot1_4))
                            and (dot3_3/abs(dot3_3) != dot3_4/abs(dot3_4))
                                ):

                            # atomic addition to prevent any counting errors
                            # if the 4 molecules are found to be in the beta-1 polymorph
                            # then 1 is added to the beta-1 index [0] in the flag array for all four molecules
                            cuda.atomic.add(flag, (frame,mol1,0), 1)
                            cuda.atomic.add(flag, (frame,mol2,0), 1)
                            cuda.atomic.add(flag, (frame,mol3,0), 1)
                            cuda.atomic.add(flag, (frame,mol4,0), 1)


                        # beta-2 check
                        if (    (dot1_1/abs(dot1_1) != dot1_2/abs(dot1_2))
                            and (dot1_1/abs(dot1_1) == dot1_3/abs(dot1_3))
                            and (dot1_1/abs(dot1_1) != dot1_4/abs(dot1_4))
                            and (dot3_3/abs(dot3_3) != dot3_4/abs(dot3_4))
                                ):

                            # atomic addition to prevent any counting errors
                            # if the 4 molecules are found to be in the beta-2 polymorph
                            # then 1 is added to the beta-2 index [1] in the flag array for all four molecules
                            cuda.atomic.add(flag, (frame,mol1,1), 1)
                            cuda.atomic.add(flag, (frame,mol2,1), 1)
                            cuda.atomic.add(flag, (frame,mol3,1), 1)
                            cuda.atomic.add(flag, (frame,mol4,1), 1)


def stretch_parallel_plot(stretch_percent,parallel_percent):
    """
    plot of % of molecules which are stretched and parallel vs time

    """

    x_axis = np.linspace(0, total_traj_time, num = int(num_frames))

    fig = plt.figure(figsize=(3.33,2.25), dpi=300)

    ax = fig.add_subplot(111)

    color1 = 'tab:blue'
    ax.plot(x_axis[1:], stretch_percent[1:], color=color1)
    color2 = 'tab:red'
    ax.plot(x_axis[1:], parallel_percent[1:], color=color2)

    ax.set_xlabel('Time (ns)', fontsize=8)
    ax.set_xlim(xmin = 0, xmax = total_traj_time)
    ax.grid(axis='x')
    plt.xticks(np.linspace(0, total_traj_time, int((total_traj_time/5)+1)), fontsize=8)

    ax.set_ylabel('Percentage of molecules', fontsize=8)
    plt.yticks(np.linspace(0, 100, 11), fontsize=8)

    ax.set_title('% of Molecules fully stretched/parallel')
    ax.legend(('Stretched','Parallel'), fontsize=8)

    plt.show()
    fig.savefig(wdir + 'stretched_parallel.jpg', format='jpg')

def nno_plot(same_indices_count_transposed):
    """
    heatmap showing how many molecules within the cut-off distance for one molecule are the same in
    subsequent frames for each molecule over time

    """

    fig2 = plt.figure(figsize=(3.33,2.25), dpi=300)
    ax2 = fig2.add_subplot(111)

    im2 = ax2.imshow(same_indices_count_transposed, aspect='auto', cmap=plt.cm.magma, extent=[0,total_traj_time,mol_count,0])
    ax2.invert_yaxis()
```

```
    ax2.set_xlabel('Time (ns)', fontsize=8)
    ax2.set_ylabel('Molecule number', fontsize=8)
    ax2.set_title('Near neighbour occupancy time')
    plt.xticks(np.linspace(0, total_traj_time, int((total_traj_time/5)+1)), fontsize=8)
    plt.yticks(np.linspace(0, mol_count, 11), fontsize=8)

    divider2 = make_axes_locatable(ax2)
    cax2 = divider2.append_axes("right", size="2%", pad=0.2)
    cbar = plt.colorbar(im2, cax=cax2, ticks=np.linspace(0,16,9))
    cbar.ax.tick_params(labelsize=8)

    fig2.savefig(wdir + 'nno.jpg', format='jpg')

def polymorph_plot(unitcell_beta1,unitcell_beta2):
    """
    plot showing the percentage of molecules in either polymorph against time

    """
    x_axis = np.linspace(0, total_traj_time, num = int(num_frames))

    fig3 = plt.figure(figsize=(3.33,2.25), dpi=300)
    ax3 = fig3.add_subplot(111)

    color1 = 'tab:blue'
    ax3.plot(x_axis[1:],unitcell_beta1[1:],color=color1)
    color2 = 'tab:red'
    ax3.plot(x_axis[1:],unitcell_beta2[1:],color=color2)
    ax3.set_xlabel('Time (ns)', fontsize=8)

    ax3.set_xlim(xmin = 0, xmax = total_traj_time)
    ax3.grid(axis='x')
    plt.xticks(np.linspace(0, total_traj_time, int((total_traj_time/5)+1)), fontsize=8)

    ax3.set_ylabel('Percentage of molecules', fontsize=8)
    plt.yticks(np.linspace(0, 100, 11), fontsize=8)

    ax3.set_title('Percentage of molecules in polymorphs')
    ax3.legend(('beta1','beta2'), fontsize=8)

    fig3.savefig(wdir + 'polymorphs.jpg', format='jpg')


### ENTER ALL REQUIRED INFORMATION TO ENABLE THE EXECUTION OF THE SCRIPT

# Working directory - this is the directory where the GROMACS trajectory (.xtc) and log (.log) files are saved

wdir = '/path/to/folder/'

TAGs = ['XXX']
phases = ['yyyyy']


start_time = time.time()

for TAG in TAGs:
    for phase in phases:
        tag_phase = tag_phases_specifics(TAG, phase)

        min_file = tag_phase[0]
        traj_file = tag_phase[1]
        log_file = tag_phase[2]

        atom_count = tag_phase[3]
        sn1_mid = tag_phase[4]
        sn1_end = tag_phase[5]
        sn2_fir = tag_phase[6]
        sn2_mid = tag_phase[7]
        sn2_end = tag_phase[8]
        sn3_mid = tag_phase[9]
        sn3_end = tag_phase[10]

        sn1end_sn3end_dist_avg = tag_phase[11]
        sn1end_sn3end_2stdev = tag_phase[12]
        sn1mid_sn3mid_dist_avg = tag_phase[13]
        sn1mid_sn3mid_2stdev = tag_phase[14]
        sn2end_sn1end_dist_avg = tag_phase[15]
        sn2end_sn1end_2stdev = tag_phase[16]
        sn2end_sn3end_dist_avg = tag_phase[17]
```

```
sn2end_sn3end_2stdev = tag_phase[18]

one_to_two_avg = tag_phase[19]
one_to_three_avg = tag_phase[20]
one_to_four_avg = tag_phase[21]
three_to_four_avg = tag_phase[22]
two_to_four_avg = tag_phase[23]
one_to_two_2stdev = tag_phase[24]
one_to_three_2stdev = tag_phase[25]
one_to_four_2stdev = tag_phase[26]
three_to_four_2stdev = tag_phase[27]
two_to_four_2stdev = tag_phase[28]

one_to_two_min = one_to_two_avg - one_to_two_2stdev
one_to_two_max = one_to_two_avg + one_to_two_2stdev
one_to_three_min = one_to_three_avg - one_to_three_2stdev
one_to_three_max = one_to_three_avg + one_to_three_2stdev
one_to_four_min = one_to_four_avg - one_to_four_2stdev
one_to_four_max = one_to_four_avg + one_to_four_2stdev
three_to_four_min = three_to_four_avg - three_to_four_2stdev
three_to_four_max = three_to_four_avg + three_to_four_2stdev
two_to_four_min = two_to_four_avg - two_to_four_2stdev
two_to_four_max = two_to_four_avg + two_to_four_2stdev



# opening the log file, searching for the timestep, number of steps and frame capture interval lines,
# extracting the values, converting them to a float and thus calculating the total trajectory time and
# number of frames in the trajectory
with open(wdir + log_file, 'r') as f:
    for line in f:
        if 'dt' in line and '=' in line:                        # finding the 'dt' line
            dt = float(re.findall("\d+\.\d+", line)[0])         # getting the timestep in ps
        if 'nsteps' in line and '=' in line:                    # finding the 'nsteps' line
            nsteps = float(re.findall("\d+\\d+", line)[0])      # getting the number of steps
            total_traj_time = nsteps * dt / 1000                # calculating the total trajectory time in ns
        if 'nstxout-compressed' in line and '=' in line:        # finding the 'nstxout-compressed' line
            frame_interval = float(re.findall("\d+\\d+", line)[0])  # getting the number of steps between each frame capture
            num_frames = int(nsteps/frame_interval) + 1         # calculating the total number of frame in the trajectory;
                                                                # add 1 to account for the extra frame which is
                                                                # added to the trajectory automatically by GROMACS
            break                                               # breaks the loop to avoid reading the whole file

# determining the number of molecules in the simulation box
mol_count = len(np.unique(np.genfromtxt(wdir + min_file, usecols=0, dtype=str, skip_header=2, skip_footer=1)))

# extracting coordinates for all atoms for all the trajectory using MDTraj
traj = xtc(wdir + traj_file,
        top = wdir + min_file)
# placing all the coordinates in a 3D numpy array with the shape=(num_frames,mol_count*atom_count,(x,y,z))
coordinates = traj.xyz
coordinates = np.ascontiguousarray(coordinates)

# calculate the % of stretched and parallel molecules
# set up all the required arrays
sn2_end_to_sn1_end_dist = np.ndarray(shape=(num_frames, mol_count), dtype=float)
sn2_end_to_sn3_end_dist = np.ndarray(shape=(num_frames, mol_count), dtype=float)
sn1_end_to_sn3_end_dist = np.ndarray(shape=(num_frames, mol_count), dtype=float)
sn1_mid_to_sn3_mid_dist = np.ndarray(shape=(num_frames, mol_count), dtype=float)
sn2_end_to_sn1_end_count = np.ndarray(shape=(num_frames))
sn2_end_to_sn3_end_count = np.ndarray(shape=(num_frames))
stretch_percent = np.zeros(shape=(num_frames))
parallel_percent = np.zeros(shape=(num_frames))

# call the function to calculate the percentage of molecules which are parallel and stretched
stretch_and_parallel(stretch_percent,
                    parallel_percent,
                    sn2_end_to_sn1_end_dist,
                    sn2_end_to_sn3_end_dist,
                    sn1_end_to_sn3_end_dist,
                    sn1_mid_to_sn3_mid_dist,
                    sn2_end_to_sn1_end_count,
                    sn2_end_to_sn3_end_count,
                    coordinates,
                    sn1_mid, sn1_end,
                    sn2_fir, sn2_mid, sn2_end,
                    sn3_mid, sn3_end,
                    sn1end_sn3end_dist_avg, sn1end_sn3end_2stdev,
                    sn1mid_sn3mid_dist_avg, sn1mid_sn3mid_2stdev,
                    sn2end_sn1end_dist_avg, sn2end_sn1end_2stdev,
```

```
                     sn2end_sn3end_dist_avg, sn2end_sn3end_2stdev,
                     atom_count)

# plot results and save file
stretch_parallel_plot(stretch_percent,parallel_percent)

print('\t', TAG, phase, 'stretch and parallel count done')



# Calculate the close neighbour occupancy number for each molecule in each frame
# Set up array to store indices of molecules which are within the cut-off distance for each molecule
# The size of the third dimension (20) is semi-arbitrary, as it is large enough to accomodaate
# the indices of all molecules found within the cut-off distance of any given molecule
cutoff_indices = np.ndarray(shape=(num_frames,mol_count,20),dtype=np.int32)
# Setting all values to -1 to facilitate comparison later
cutoff_indices[:,:,:] = -1
# Call function
cutoff_indices_1nm(cutoff_indices,coordinates,sn2_mid,atom_count)
# Set array to count all similar molecule indices between frames
same_indices_count = np.zeros(shape=(num_frames,mol_count))
# Call function
same_indices(same_indices_count,cutoff_indices)
# Transpose the array for plotting purposes (frame(time) on the x-axis)
same_indices_count_transposed = np.transpose(same_indices_count)

# plot results and save file
nno_plot(same_indices_count_transposed)

print('\t', TAG, phase, 'near neighbour occupancy count done')


# Determine the percentage of molecules in a specific polymorph
# Set up array to act as counter for each time a molecule is found to be in either polymorph
# 3rd dimension[0] = beta 1, 3rd dimension[1] = beta 2
# Array can be large and is hence a memory-managed array
flag = cuda.managed_array(shape=(num_frames,mol_count,2), dtype=np.int32)
# Set all values of array to 0
flag[:,:,:] = 0

# The polymorphic determination is done 1 frame at a time for GPU memory management purposes
for frame in range(num_frames):

    st = time.time()
    # set up GPU synchronisation settings
    stream = cuda.stream()
    start = cuda.event()
    stop = cuda.event()
    cuda.synchronize()
    start.record(stream=stream)

    # set up 3D GPU kernel
    threadsperblock = (4,4,4)
    blockspergrid_x = math.ceil(mol_count / threadsperblock[0])
    blockspergrid_y = math.ceil(mol_count / threadsperblock[1])
    blockspergrid_z = math.ceil(mol_count / threadsperblock[2])
    blockspergrid = (blockspergrid_x, blockspergrid_y, blockspergrid_z)

    # call function
    unit_cell_determination[blockspergrid, threadsperblock, stream](
        frame,
        coordinates,
        flag,
        sn1_end,
        sn2_fir,
        sn2_mid,
        sn2_end,
        atom_count,
        one_to_two_min,
        one_to_two_max,
        one_to_three_min,
        one_to_three_max,
        one_to_four_min,
        one_to_four_max,
        three_to_four_min,
        three_to_four_max,
        two_to_four_min,
        two_to_four_max)
```

```
            # stop synchronisation
            stop.record(stream=stream)

            # dummy for loop to allow all processes to finish and thus avoid synchronisation issues
            counter = 0
            while not stop.query():
                counter += 1


        # set up array to store whether a molecule is found in either polymorph
        # 3rd dimension[0] = beta 1, 3rd dimension[1] = beta 2
        beta1_beta2_ratio = np.zeros(shape=(num_frames,mol_count,2), dtype=int)

        for frame in range(num_frames):
            for num in range(mol_count):
                if flag[frame,num,0] > flag[frame,num,1]:
                    beta1_beta2_ratio[frame,num,0] = 1
                    beta1_beta2_ratio[frame,num,1] = 0
                elif flag[frame,num,0] < flag[frame,num,1]:
                    beta1_beta2_ratio[frame,num,0] = 0
                    beta1_beta2_ratio[frame,num,1] = 1
                elif flag[frame,num,0] == 0 and flag[frame,num,1] == 0:
                    beta1_beta2_ratio[frame,num,0] = 0
                    beta1_beta2_ratio[frame,num,1] = 0
                elif flag[frame,num,0] == flag[frame,num,1]:
                    beta1_beta2_ratio[frame,num,0] = 0.5
                    beta1_beta2_ratio[frame,num,1] = 0.5

        # sum up number of molecules found to be in either polymorph and calculate percentage
        unitcell_beta1 = np.zeros(shape=(num_frames), dtype=np.int32)
        unitcell_beta2 = np.zeros(shape=(num_frames), dtype=np.int32)

        for frame in range(num_frames):
            unitcell_beta1[frame] = beta1_beta2_ratio[frame,:,0].sum()/mol_count*100
            unitcell_beta2[frame] = beta1_beta2_ratio[frame,:,1].sum()/mol_count*100

        # plot results and save file
        polymorph_plot(unitcell_beta1,unitcell_beta2)

        print('\t', TAG, phase, 'polymorph count done')


print('This script took',
      '{:.2f}'.format((time.time()-start_time)), 'seconds or',
      '{:.2f}'.format((time.time()-start_time)/60), 'minutes or',
      '{:.2f}'.format((time.time()-start_time)/3600), 'hours', '\n')
```
